# Supplementary material for: Cross-sectional associations between 24-hour time-use composition, grey matter volume and cognitive function in healthy older adults
Source: Int J Behav Nutr Phys Act. 2024 Jan 30;21:11. doi: 10.1186/s12966-023-01557-4 (PMC10829181; doi:10.1186/s12966-023-01557-4)
Supplement: Supplementary file 1 — Supplementary Material 1: Strengthening the reporting of observational studies in epidemiology (STROBE) checklist [file 12966_2023_1557_MOESM1_ESM.docx]

STROBE Statement—checklist of items that should be included in reports of observational studies

|  | Item No. | Recommendation | Page  No. | Relevant text from manuscript |
| --- | --- | --- | --- | --- |
| **Title and abstract** | 1 | (*a*) Indicate the study’s design with a commonly used term in the title or the abstract | 1 | **Title:** Cross-sectional associations between 24-hour time-use composition, grey matter volume and cognitive function in healthy older adults. |
|  |  | (*b*) Provide in the abstract an informative and balanced summary of what was done and what was found | 2 | Lines 29-44: This cross-sectional study included 378 older adults (65.6 ± 3.0 years old, 123 male) from the ACTIVate study across two Australian sites (Adelaide and Newcastle). Time-use composition was captured using 7-day accelerometry, and T1-weighted magnetic resonance imaging was used to measure grey matter volume both globally and across regions of interest (ROI: frontal lobe, temporal lobe, hippocampi, and lateral ventricles). Pairwise correlations were used to explore univariate associations between time-use variables, grey matter volumes and cognitive outcomes. Compositional data analysis linear regression models were used to quantify associations between ROI volumes and time-use composition, and explore potential associations between the interaction between ROI volumes and time-use composition with cognitive outcomes. Results: After adjusting for covariates (age, sex, education), there were no significant associations between time-use composition and any volumetric outcomes. There were significant interactions between time-use composition and frontal lobe volume for long-term memory (p=0.018) and executive function (p=0.018), and between time-use composition and total grey matter volume for executive function (p=0.028). Spending more time in moderate-vigorous PA was associated with better long-term memory scores, but only for those with smaller frontal lobe volume (below the sample mean). Conversely, spending more time in sleep and less time in sedentary behaviour was associated with better executive function in those with smaller total grey matter volume |
| Introduction | | | |  |
| Background/rationale | 2 | Explain the scientific background and rationale for the investigation being reported | 3-5 | *Please see lines 53-112 for scientific background and rationale for the study.* |
| Objectives | 3 | State specific objectives, including any prespecified hypotheses | 5 | Lines 115-120: “To address these important gaps in knowledge, cross-sectional data from the baseline phase of the ACTIVate study were used to investigate (a) whether 24-hour time-use composition is associated with grey matter volume, and (b) whether grey matter volume moderates the relationship between 24-hour time-use composition and cognitive function.”. |
| Methods | | | |  |
| Study design | 4 | Present key elements of study design early in the paper | 5 | Lines 116-117: “To address these important gaps in knowledge, cross-sectional data from the baseline phase of the ACTIVate study was used to investigate …” |
| Setting | 5 | Describe the setting, locations, and relevant dates, including periods of recruitment, exposure, follow-up, and data collection | 6, 7 | Line 133: “Participants were recruited for the ACTIVate study using a rolling convenience sampling strategy (29)”. ***please note that this sentence refers the reader to the protocol paper for the larger study which contains further details of these criteria.*  Line 143: “Data were collected between August 2020 and February 2022”.  Lines 163-164: “MRI acquisition was performed on a Siemens Skyra 3T scanner in Adelaide, and a Siemens Prisma 3T scanner in Newcastle, both using ….” |
| Participants | 6 | (*a*) *Cohort study*—Give the eligibility criteria, and the sources and methods of selection of participants. Describe methods of follow-up  *Case-control study*—Give the eligibility criteria, and the sources and methods of case ascertainment and control selection. Give the rationale for the choice of cases and controls  *Cross-sectional study*—Give the eligibility criteria, and the sources and methods of selection of participants | 6 | Lines 129-136: “Eligibility criteria for the ACTIVate study have been reported in detail elsewhere (29). Briefly, participants were eligible if they were aged 60-70 years, fluent in English, had no clinical diagnoses of dementia or any other neurological or psychiatric disorders, did not have an intellectual or major physical disability, and presented no contraindications to transcranial magnetic stimulation or MRI screening tools (30).  Participants were recruited for the ACTIVate study using a rolling convenience sampling strategy (29). Those who met initial eligibility criteria were further screened against cognitive impairment using the Montreal Cognitive Assessment (blind) via phone interview. Participants who scored <13 (out of a potential 22) were excluded from the study.”  Line 142: “Data were collected between August 2020 and February 2022”.  ***please note that this section refers the reader to the protocol paper for the larger study which contains further details of these criteria.* |
|  |  | (*b*) *Cohort study*—For matched studies, give matching criteria and number of exposed and unexposed  *Case-control study*—For matched studies, give matching criteria and the number of controls per case |  |  |
| Variables | 7 | Clearly define all outcomes, exposures, predictors, potential confounders, and effect modifiers. Give diagnostic criteria, if applicable | 6-8 | ***Please see section 2.3 (lines 142-192) which outlines study measures used in this study (including the following sections: 2.3.1 Device-measured time-use patterns; 2.3.2 Brain imaging and MRI processing; 2.3.3. Cognitive function measures; 2.3.4 Covariates).* |
| Data sources/ measurement | 8* | For each variable of interest, give sources of data and details of methods of assessment (measurement). Describe comparability of assessment methods if there is more than one group | 6-8 | ***Please see section 2.3 (lines 142-192) which outlines study measures used in this study (including the following sections: 2.3.1 Device-measured time-use patterns; 2.3.2 Brain imaging and MRI processing; 2.3.3. Cognitive function measures; 2.3.4 Covariates).* |
| Bias | 9 | Describe any efforts to address potential sources of bias | - | - |
| Study size | 10 | Explain how the study size was arrived at | 6 | Lines 132-136: “Power calculations were used to determine the required sample size for the larger ACTIVate study (based on cognitive outcomes), which have been detailed extensively elsewhere (29). Briefly, aiming for 80% power, allowing for attrition and response rate at recruitment and accounting for the longitudinal design of the study, the final sample size of 448 participants was determined.”. |

| Quantitative variables | 11 | Explain how quantitative variables were handled in the analyses. If applicable, describe which groupings were chosen and why | 6-8 | ***Please see section 2.3 (lines 142-192) which outlines handling of quantitative variables (including the following sections: 2.3.1 Device-measured time-use patterns; 2.3.2 Brain imaging and MRI processing; 2.3.3. Cognitive function measures; 2.3.4 Covariates).* |
| --- | --- | --- | --- | --- |
| Statistical methods | 12 | (*a*) Describe all statistical methods, including those used to control for confounding | 9-11 | ***Please see section 2.4 (lines 194-267) which provides a detailed overview of statistical analysis (including the following sections: 2.4.1 Pairwise correlations; 2.4.2 Compositional data analysis (CoDA); 2.4.3 Modelling reallocations of time).* |
|  |  | (*b*) Describe any methods used to examine subgroups and interactions |  |  |
|  |  | (*c*) Explain how missing data were addressed | 12 | Lines 270-275: “Of the original 426 participants recruited in the baseline phase of the ACTIVate study, 395 participants completed both T1 MPRAGE and T2 FLAIR imaging protocols. Seventeen participants were removed from the dataset as they did not have valid accelerometry data: 7 did not meet minimum criteria for a valid accelerometry dataset (i.e., less than minimum required days of recording); 8 were missing accelerometry data; and two had >1500 minutes of recorded time use per day. The overall final sample included 378 older adults (65.6 ± 3.0 years old, 123 males)”. |
|  |  | (*d*) *Cohort study*—If applicable, explain how loss to follow-up was addressed  *Case-control study*—If applicable, explain how matching of cases and controls was addressed  *Cross-sectional study*—If applicable, describe analytical methods taking account of sampling strategy |  | N/A |
|  |  | (*e*) Describe any sensitivity analyses |  | N/A |
| Results | | | | |
| Participants | 13* | (a) Report numbers of individuals at each stage of study—eg numbers potentially eligible, examined for eligibility, confirmed eligible, included in the study, completing follow-up, and analysed | 12 | Lines 270-275: “Of the original 426 participants recruited in the baseline phase of the ACTIVate study, 395 participants completed both T1 MPRAGE and T2 FLAIR imaging protocols. Seventeen participants were removed from the dataset as they did not have valid accelerometry data: 7 did not meet minimum criteria for a valid accelerometry dataset (i.e., less than minimum required days of recording); 8 were missing accelerometry data; and two had >1500 minutes of recorded time use per day. The overall final sample included 378 older adults (65.6 ± 3.0 years old, 123 males)”. |
|  |  | (b) Give reasons for non-participation at each stage |  |  |
|  |  | (c) Consider use of a flow diagram | - | - |
| Descriptive data | 14* | (a) Give characteristics of study participants (eg demographic, clinical, social) and information on exposures and potential confounders | 12-13 | ***Please see Table 1: Participant demographics.*  Lines 274-279: “The overall final sample included 378 older adults (65.6 ± 3.0 years old, 123 males). Means, standard deviations and range (minimum and maximum) of key continuous variables are presented in Table 1. Participants had low white matter hyperintensity burden (mean = 2 ml) and were highly active, spending approximately 4.5 hours per day in physical activity (1.5 hours in MVPA; 3 hours in LPA), 11.1 hours in sedentary behaviour, and 8.4 hours sleeping. Participants’ time-use compositions are displayed in Figure 1”. |
|  |  | (b) Indicate number of participants with missing data for each variable of interest | 17 | Caption for Table 4, lines 357-361: “Sample sizes for each cognitive outcome varied due to missing data: long-term memory, n=360; executive function, n=363; processing speed, n=368”. |
|  |  | (c) *Cohort study*—Summarise follow-up time (eg, average and total amount) | - | N/A |
| Outcome data | 15* | *Cohort study*—Report numbers of outcome events or summary measures over time | - | N/A |
|  |  | *Case-control study—*Report numbers in each exposure category, or summary measures of exposure | *-* | N/A |
|  |  | *Cross-sectional study—*Report numbers of outcome events or summary measures | 12 | Lines 270-279: “Of the original 426 participants recruited in the baseline phase of the ACTIVate study, 395 participants completed both T1 MPRAGE and T2 FLAIR imaging protocols. Seventeen participants were removed from the dataset as they did not have valid accelerometry data: 7 did not meet minimum criteria for a valid accelerometry dataset (i.e., less than minimum required days of recording); 8 were missing accelerometry data; and two had >1500 minutes of recorded time use per day. The overall final sample included 378 older adults (65.6 ± 3.0 years old, 123 males). Means, standard deviations and range (minimum and maximum) of key continuous variables are presented in Table 1. Participants had low white matter hyperintensity burden (mean = 2 ml) and were highly active, spending approximately 4.5 hours per day in physical activity (1.5 hours in MVPA; 3 hours in LPA), 11.1 hours in sedentary behaviour, and 8.4 hours sleeping. Participants’ time-use compositions are displayed in Figure 1”. |
| Main results | 16 | (*a*) Give unadjusted estimates and, if applicable, confounder-adjusted estimates and their precision (eg, 95% confidence interval). Make clear which confounders were adjusted for and why they were included | 8, 13-17 | Lines 188-190: “Age (years), sex (male, female) and education (total years) were entered as covariates in linear regression models, based on previous evidence of their associations with grey matter volume (39-41) and cognitive function (3, 42)”.  ***Please note that all linear regression models were adjusted for age, sex and education, and p-values were further adjusted for false discovery rate (page 13-17)* |
|  |  | (*b*) Report category boundaries when continuous variables were categorized | 20, 21 | Lines 426-427: “Mean frontal lobe volume (corrected) in the ‘upper’ group = 174.8 ± 5.0, range = 168.3, 193.0. Mean frontal lobe volume (corrected) in the ‘lower’ group = 161.2 ± 5.0, range = 143.3, 168.3”.  Lines 435-436: “Mean total grey matter volume (corrected) in the ‘upper’ group = 611.4 ± 10.6, range = 596.7, 647.9. Mean total grey matter volume (corrected) in the ‘lower’ group = 580.7 ± 12.9, range = 519.8, 596.7.”. |
|  |  | (*c*) If relevant, consider translating estimates of relative risk into absolute risk for a meaningful time period | - | N/A |

| Other analyses | 17 | Report other analyses done—eg analyses of subgroups and interactions, and sensitivity analyses | 17-18 | Lines 363-417: “To further investigate these interactions, we plotted a series of model-estimated cognitive response curves which demonstrate the estimated associations of time reallocations with long-term memory and executive function outcomes, across high and low brain volume groups in the frontal lobe and total grey matter, respectively. Additionally, post-hoc multiple linear regression analyses between time-use composition and each cognitive outcome within high and low volume brain volume groups can be viewed in Additional File 2. High and low volume groups were quantified as those above and below the mean frontal lobe volume (168ml) and total grey matter volume (596ml) in the sample, as the data were normally distributed (therefore a median split achieved similar data separation). Before creating the response plots, regression models containing the significant interactions were replicated with frontal lobe volume and total grey matter volume included as categorical variables (two levels, upper and lower volume group, rather than as a continuous variable) to ensure that the interaction between time-use composition and each ROI volume remained significant. Interestingly, the interaction between time-use composition and frontal lobe volume (as a categorical variable) did not remain significant for executive function (padj=0.80). This remained true when frontal lobe volume was split into quartiles (padj=0.31). For this reason, the time-use composition by frontal lobe volume interaction for the executive function outcome was not further explored here. Figures 2 and 3 display the predicted differences in long-term memory z-score and executive function z-score associated with reallocations of time from the reference mean time-use composition towards and away from each time-use behaviour (positive and negative reallocations on the x-axis), whilst drawing time spent in the remaining behaviours in the 24-hour day pro-rata (i.e., one-to-remaining reallocations). One-for-one reallocation models are displayed in Additional File 1. To illustrate how brain structure interacts with these relationships, predictions were plotted separately for those above and below the mean frontal lobe and total grey matter volume, respectively.  Figure 2 (displaying one-for-remaining reallocations) suggests that for those with smaller frontal lobe volume (below the sample mean), more time in MVPA was associated with better long-term memory performance. One-for-one reallocation plots (Supplementary Figure 1, Additional File 1) supported that this reallocation was most beneficial when time was taken from either sleep or sedentary behaviour, whilst taking time from LPA had positive but non-significant associations with long-term memory. Contrary to this, reallocating time towards MVPA had little predicted benefit for those with greater frontal lobe volume (above the sample mean). Spending more time in LPA at the equal expense of other behaviours (Figure 2) was associated with small unfavorable differences in long-term memory performance for those with smaller frontal lobe volume, and slight but favorable differences in performance for those with greater frontal lobe volume. Finally, reallocating time towards or away from sleep and sedentary behaviour (at the equal expense of remaining behaviours) had similar (minimal) associations with long-term memory performance for both high and low frontal lobe volume groups. However, one-for-one reallocations suggested that for those with greater frontal lobe volume, increasing time in sedentary behaviour at the expense of sleep was positively associated with long-term memory.  Figure 3 shows that the executive function response curves for sleep and sedentary behaviour reallocations differ by total grey matter volume. Although supplementary regression analyses demonstrated that there were no statistically significant associations between time-use composition and executive function within total grey matter volume groups (Additional File 2), the data are described further here for completeness. Figure 3 suggests that for those with smaller total grey matter volume, more time in sleep and less time in sedentary behaviour was favorably associated with executive function, while these reallocations were negatively associated with executive function in those with greater total grey matter volume. One-for-one reallocations (Supplementary Figure 2, Additional File 1) confirmed that increasing time in sleep at the direct expense of sedentary behaviour was beneficial for executive function in those with smaller total grey matter volume, whilst no other reallocations towards sleep were associated with executive function (i.e., from LPA or MVPA). Reallocating time towards or away from LPA had minimal associations with executive function across both high and low total grey matter volume groups. This was consistently observed across both proportional and one-for-one reallocations (i.e., regardless of which other compositional part time was reallocated from or towards). Finally, spending more time in MVPA at the equal expense of all other behaviours had small unfavorable associations with executive function in the higher total grey matter volume group, whilst reallocations towards or away from MVPA had minimal associations in the lower total grey matter group. One-for-one swaps supported these observations, suggesting that increasing or decreasing time in MVPA at the expense of sleep, LPA or sedentary behaviour had minimal associations with executive function. |
| --- | --- | --- | --- | --- |
| Discussion | | | | |
| Key results | 18 | Summarise key results with reference to study objectives | 22, 25 | Lines 440-444: “The primary aim of this study was to explore whether 24-hour time-use composition of MVPA, LPA, sedentary behaviour and sleep was associated with total and regional grey matter volume (temporal lobe, frontal lobe, hippocampus, and lateral ventricle volume) in healthy older adults. Our main finding was that there were no associations between 24-hour time-use composition and any volumetric outcomes, after adjustment for age, sex, and education”.  Lines 520-529: “Several mechanisms likely underlie the relationship between lifestyle and cognitive function in older adults, including maintenance of grey matter volume. This relationship has only been investigated when considering physical activity, sedentary behaviour and sleep independently, rather than as a 24-hour composition. In our secondary analysis, we found that long-term memory was associated with the interaction between 24-hour time-use composition and frontal lobe volume, and that executive function was associated with the interaction between 24-hour time-use composition and both total grey matter volume and frontal lobe volume (although, the frontal lobe by executive function interaction was not further explored). It should be noted that after separating the dataset by mean frontal lobe and total grey matter volumes (Additional File 2), only the association between time-use composition and long-term memory in the smaller frontal lobe sub-group remained statistically significant”. |
| Limitations | 19 | Discuss limitations of the study, taking into account sources of potential bias or imprecision. Discuss both direction and magnitude of any potential bias | 28-29 | Lines 594-624: “However, there are several limitations that should be noted. As outlined in a previous study using the same dataset (28), the recruited sample were highly active and highly educated despite best efforts to recruit participants across a variety of activity and dietary patterns. The cross-sectional nature of the study limits the inferences that can be made about causal relationships between variables. Due to the exploratory nature of the study, a number of additional modifiable dementia risk factors and other important indicators of health status which may relate to inter-individual variability in time use, brain volume and cognitive function were not included as covariates in this study (e.g., adiposity, depression, smoking, alcohol consumption) or were not measured as part of the wider ACTIVate study (e.g., aerobic fitness). Thus, observed associations may have been attenuated by accounting for other health outcomes and these should be considered in future research. There are a number of limitations associated with the use of wrist-worn accelerometers to delineate time-use behaviours which should be acknowledged. Wrist-worn accelerometers are less sensitive in detecting sitting time compared to other wear locations (e.g., thigh) as they are unable to differentiate lower body postures (e.g., sitting versus standing), and so it is possible that periods of standing were incorrectly classified as sedentary time rather than LPA, and on the contrary, it is possible that high intensity activities such as stationary cycling may have been captured as sedentary behaviour due to the limited movement of the upper limb during the activity. The cut points used to differentiate time-use behaviours in this study were derived from a validation study conducted in a sample of younger adults, and thus the distribution of time spent in different intensity bands in this sample may differ in older adults. Taken together, it is possible that the use of wrist-worn accelerometers and the chosen cut points resulted in an overestimation of sedentary time, and therefore the findings surrounding sedentary behaviour should be interpreted with caution. Finally, the handling of non-wear time and subsequent imputation methods (i.e., proportional re-scaling of time-use data across all components using closure function) may have resulted in the over-estimation of time spent in sleep. Non-wear time is not typically accrued during sleep, and thus increasing time in sleep as a result of the proportional rescaling across all time-use components may have resulted in the overestimation of time spent in this behaviour (see Haszard et al. (58) for applied example).”. |
| Interpretation | 20 | Give a cautious overall interpretation of results considering objectives, limitations, multiplicity of analyses, results from similar studies, and other relevant evidence | 22-29 | Lines 440-518: *Please see these paragraphs which aim to interpret and contextualise the findings of our primary aim/analysis.*  Lines 520-592: *Please see these paragraphs which aim to interpret and contextualise the findings of our secondary aim/analysis.* |
| Generalisability | 21 | Discuss the generalisability (external validity) of the study results | 26-27 | Lines 584-592: “Importantly, results in these secondary analyses were derived from cross-sectional data and the predicted associations were modest in scale, and therefore should be interpreted with some caution. Despite correcting each ROI volume for total intracranial volume in this study, we cannot deduce that smaller ROI volumes reflect accelerated atrophy due to the cross-sectional nature of the study. If each of these findings are upheld in longitudinal studies, they may indicate that lifestyle interventions which aim to maintain or improve cognitive functions such as memory and executive function (as a means to reduce dementia risk or delay dementia onset) could be tailored based on individual differences in brain volume (i.e., prescribing higher intensity physical activity for those with more progressed brain atrophy in the frontal lobe, or targeting sleep duration in those with more progressed total grey matter atrophy)”. |
| Other information | |  | | |
| Funding | 22 | Give the source of funding and the role of the funders for the present study and, if applicable, for the original study on which the present article is based | 30-31 | Lines 582-588: “MM was supported by a Dementia Australia Research Foundation PhD scholarship. The ACTIVate study is funded by an NHMRC Boosting Dementia Research Priority Round 5 grant (GNT1171313, $1.23m). DD was supported by an NHMRC Early Career Fellowship (GNT1162166, 2019-2022) and an ARC Discovery Early Career Award (DE230101174, 2023-2025). MG was supported by an Australian Research Council (ARC) fellowship (DE200100575). TS was supported by a Hospital Research Foundation grant (C-PJ-008-Transl-2020) awarded to AS and DD. AS was supported by a Dementia Australia Henry Brodaty Mid-Career Fellowship. The funding bodies were not involved the design or conduct of the study; collection, analysis, and interpretation of the data; preparation, review, or approval of the manuscript; or the decision to submit the manuscript for peer-reviewed publication”. |

*Give information separately for cases and controls in case-control studies and, if applicable, for exposed and unexposed groups in cohort and cross-sectional studies.
