# Supplementary material for: Cross-sectional associations between 24-hour time-use composition, grey matter volume and cognitive function in healthy older adults
Source: Int J Behav Nutr Phys Act. 2024 Jan 30;21:11. doi: 10.1186/s12966-023-01557-4 (PMC10829181; doi:10.1186/s12966-023-01557-4)
Supplement: Supplementary file 2 — Supplementary Material 2: Additional file 1 (Supplementary Figures 1 and 2; one-for-one reallocation modelling) [file 12966_2023_1557_MOESM2_ESM.docx]

**Additional File 1**


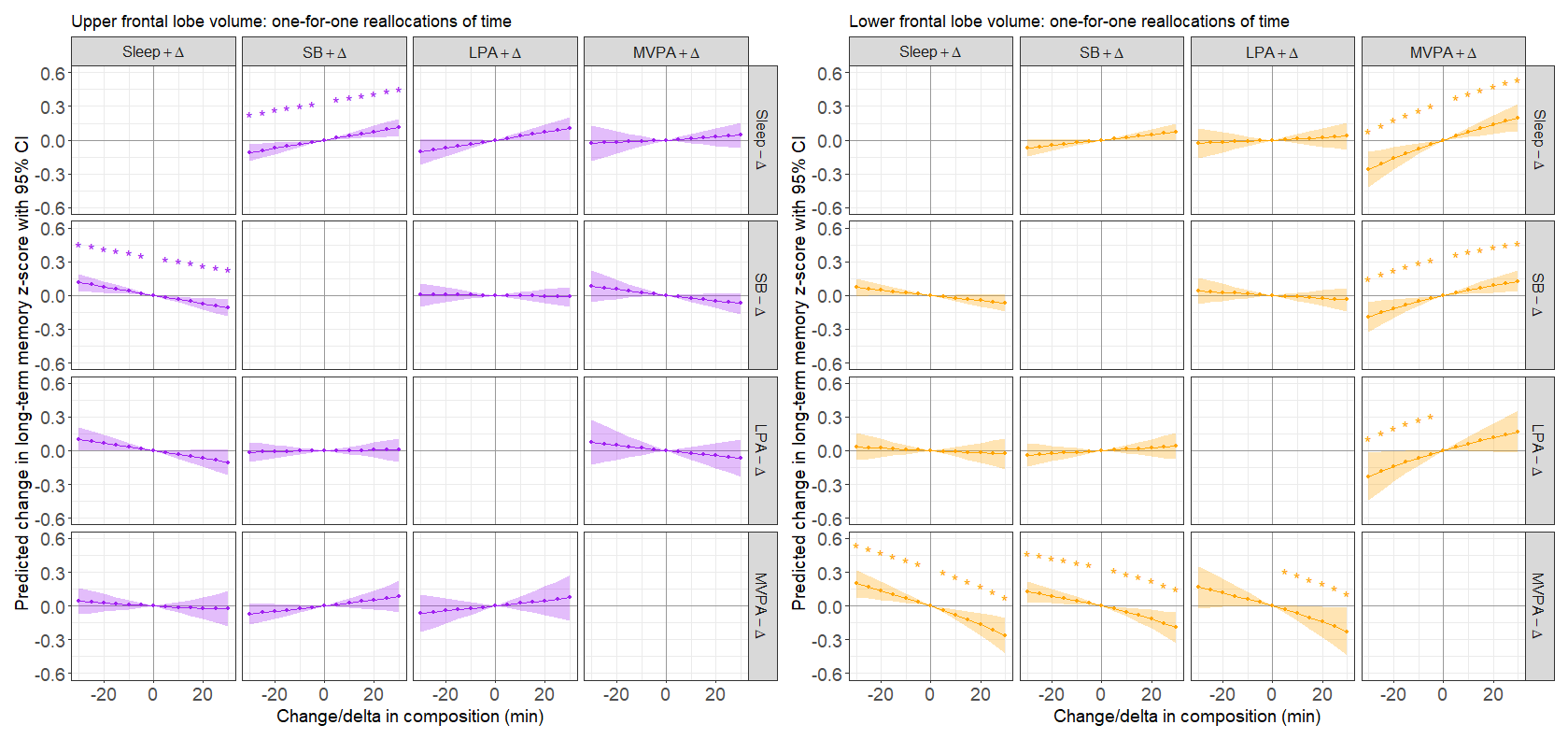
**Supplementary Figure 1.** One-for-one reallocations of time between time-use behaviours, and their associations with long-term memory outcomes. Reallocations are plotted separately for those above (left panel, in purple) and below (right panel, in orange) the mean frontal lobe volume. Each cell displays the predicted long-term memory z-score (y-axis) associated with a change in the behaviour listed in the header (top of cell) at the expense of the behaviour listed on the right of the cell, in 5-minute increments (x-axis). Asterisks (*) indicate reallocations that are statistically significant (i.e., 95% CI does not include zero).


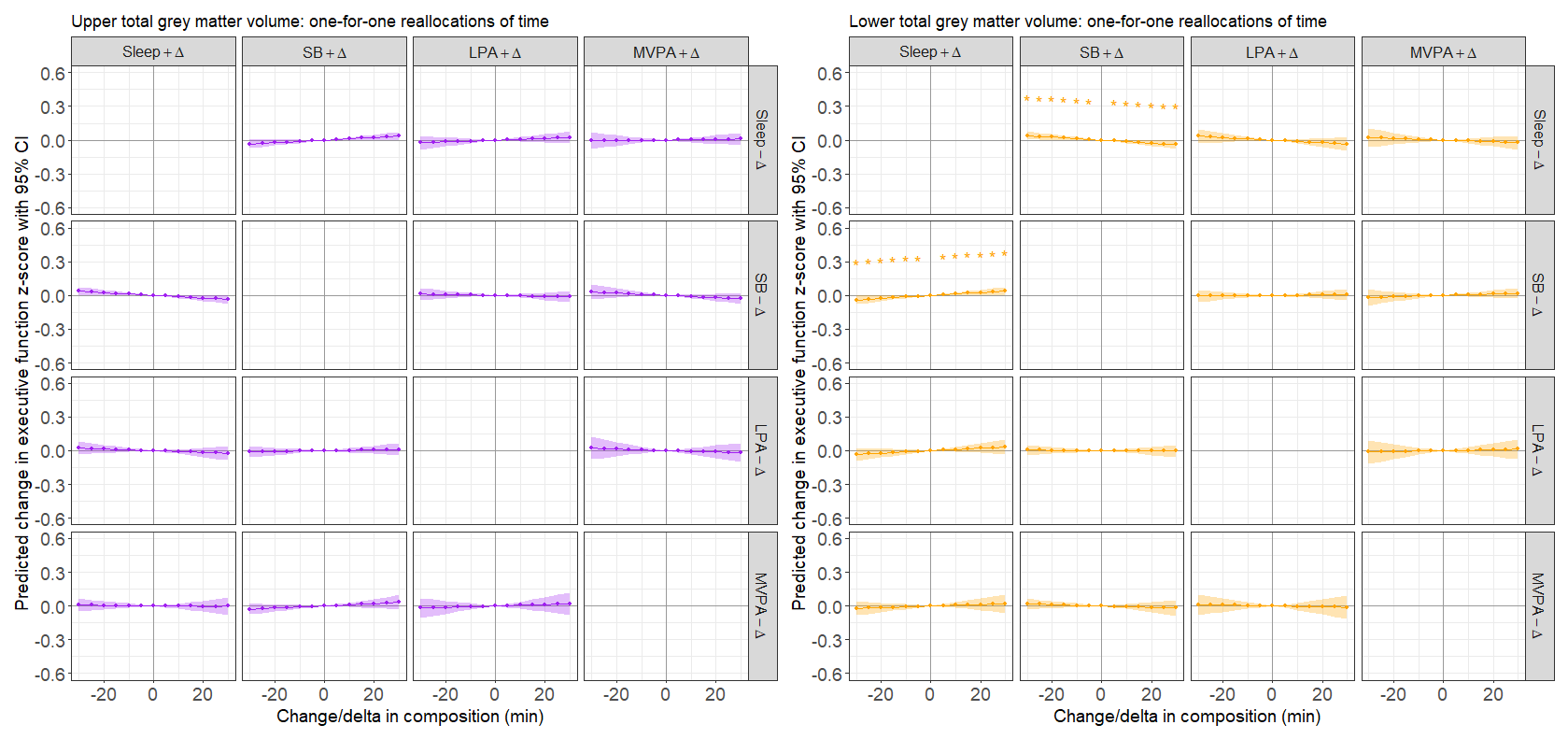
**Supplementary Figure 2.** One-for-one reallocations of time between time-use behaviours, and their associations with executive function outcomes. Reallocations are plotted separately for those above (left panel, in purple) and below (right panel, in orange) the mean total grey matter volume. Each cell displays the predicted executive function z-score (y-axis) associated with a change in the behaviour listed in the header (top of cell) at the expense of the behaviour listed on the right of the cell, in 5-minute increments (x-axis). Asterisks (*) indicate reallocations that are statistically significant (i.e., 95% CI does not include zero).
