# Supplementary material for: Cross-sectional associations between 24-hour time-use composition, grey matter volume and cognitive function in healthy older adults
Source: Int J Behav Nutr Phys Act. 2024 Jan 30;21:11. doi: 10.1186/s12966-023-01557-4 (PMC10829181; doi:10.1186/s12966-023-01557-4)
Supplement: Supplementary file 3 — Supplementary Material 3: Additional file 2 (Supplementary analyses within volumetric sub-groups) [file 12966_2023_1557_MOESM3_ESM.docx]

**Additional File 2.**

|  | | **Long-term memory** | | | | | | **Executive function** | | | | | |
| --- | --- | --- | --- | --- | --- | --- | --- | --- | --- | --- | --- | --- | --- |
|  |  | **Higher frontal lobe vol** | | | **Lower frontal lobe vol** | | | **Higher total GM vol** | | | **Lower total GM vol** | | |
| **Model** | **Variable** | **F** | ***p*** | ***adj.p*** | **F** | ***p*** | ***adj.p*** | **F** | ***p*** | ***adj.p*** | **F** | ***p*** | ***adj.p*** |
| 1 | Age | 0.29 | 0.591 | 0.887 | 2.79 | 0.096 | 0.145 | 2.92 | 0.089 | 0.089 | 17.28 | <0.001 | <0.001 |
|  | Sex | 2.11 | 0.149 | 0.446 | 0.27 | 0.604 | 0.604 | 9.16 | 0.003 | 0.089 | 5.41 | 0.021 | **0.031** |
|  | Education | 0.01 | 0.927 | 0.927 | 5.39 | 0.021 | 0.064 | 3.02 | 0.084 | 0.089 | 0.92 | 0.338 | 0.338 |
| 2 | Age | 1.19 | 0.278 | 0.367 | 1.27 | 0.262 | 0.262 | 2.39 | 0.124 | 0.164 | 16.61 | <0.001 | **<0.001** |
|  | Sex | 2.04 | 0.155 | 0.311 | 1.66 | 0.199 | 0.262 | 9.71 | 0.002 | **0.008** | 5.51 | 0.020 | **0.040** |
|  | Education | 0.60 | 0.440 | 0.440 | 5.50 | 0.020 | **0.040** | 2.61 | 0.107 | 0.164 | 1.66 | 0.199 | 0.204 |
|  | Time-use composition | 3.00 | 0.032 | 0.127 | 3.95 | 0.009 | **0.037** | 1.72 | 0.164 | 0.164 | 1.54 | 0.204 | 0.204 |

Supplemental analysis of associations between time-use composition and cognitive outcomes within brain volume sub-groups.

*Note.* F=F statistic; *adj.p* = p-value adjusted for false discovery rate; vol = volume (ml); GM = grey matter. Bold denotes p-values that remained statistically significant after false discovery rate adjustment (*p*<0.05). Mean frontal lobe volume (corrected) in the ‘upper’ group = 174.8 ± 5.0, range = 168.3, 193.0. Mean frontal lobe volume (corrected) in the ‘lower’ group = 161.2 ± 5.0, range = 143.3, 168.3. Mean total grey matter volume (corrected) in the ‘upper’ group = 611.4 ± 10.6, range = 596.7, 647.9. Mean total grey matter volume (corrected) in the ‘lower’ group = 580.7 ± 12.9, range = 519.8, 596.7.
